# Supplementary material for: Precise Molecular Engineering of Type I Photosensitizer with Aggregation-Induced Emission for Image-Guided Photodynamic Eradication of Biofilm
Source: Molecules. 2023 Jul 12;28(14):5368. doi: 10.3390/molecules28145368 (PMC10385678; doi:10.3390/molecules28145368)
Supplement: Supplementary file 1 [file molecules-28-05368-s001.zip › molecules-2350494-supplementary.pdf]

Supporting Information

Precise Molecular Engineering of Type I Photosensitizer with Aggregation-Induced Emission for Image-Guided Photodynamic Eradication of Biofilm

Jinghong Shi[a#], Yucheng Wang[b#], Wei He[ac], Ziyue Ye[b], Mengli Liu[b], Zheng Zhao[bc], Jacky Wing Yip Lam[a], Pengfei Zhang[d], Ryan Tsz Kin Kwok[ac]*, Ben Zhong Tang[abc]*

a. Department of Chemistry, Hong Kong Branch of Chinese National Engineering Research Center for Tissue Restoration and Reconstruction, The Hong Kong University of Science and Technology, Clear Water Bay, Kowloon, Hong Kong, China.

b. School of Science and Engineering, Shenzhen Key Laboratory of Functional Aggregate Materials, The Chinese University of Hong Kong, Shenzhen, Guangdong 518172, China.

c. HKUST-Shenzhen Research Institute, South Area Hi-Tech Park, Nanshan, Shenzhen, Guangdong Province 518057

d. Shenzhen Key Laboratory for Molecular Imaging, CAS Key Lab for Health Informatics, Shenzhen Institutes of Advanced Technology, Chinese Academy of Sciences, Shenzhen 518055, China

* Correspondence: chryan@ust.hk (R.T.K. Kwok); tangbenz@cuhk.edu.cn (B.Z. Tang)

Synthesis of TTTDM

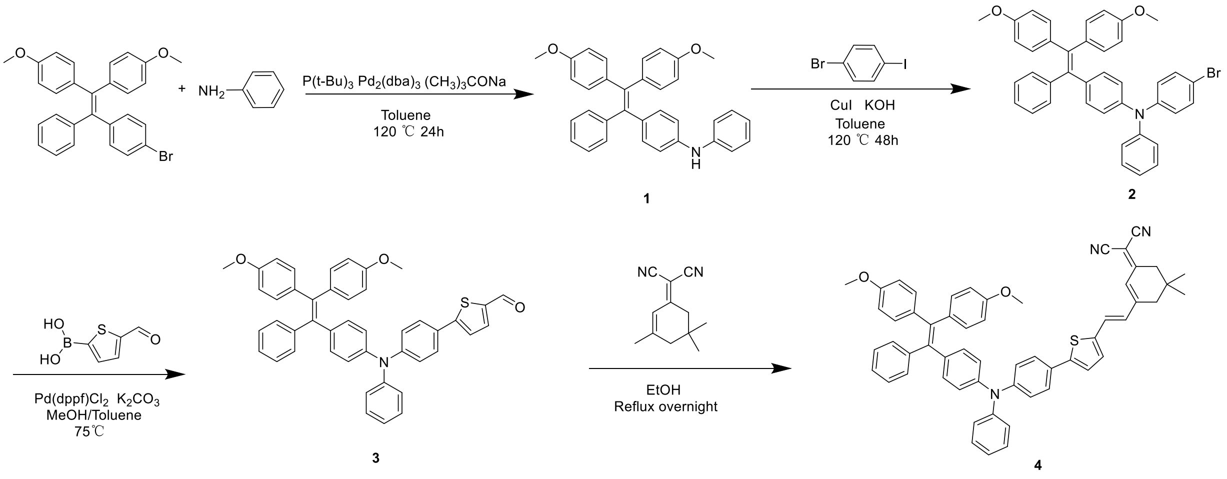

Scheme S1. Synthesis of Type-I photosensitizer TTTDM.

^{1}H NMR Spectra of intermediate and TTTDM

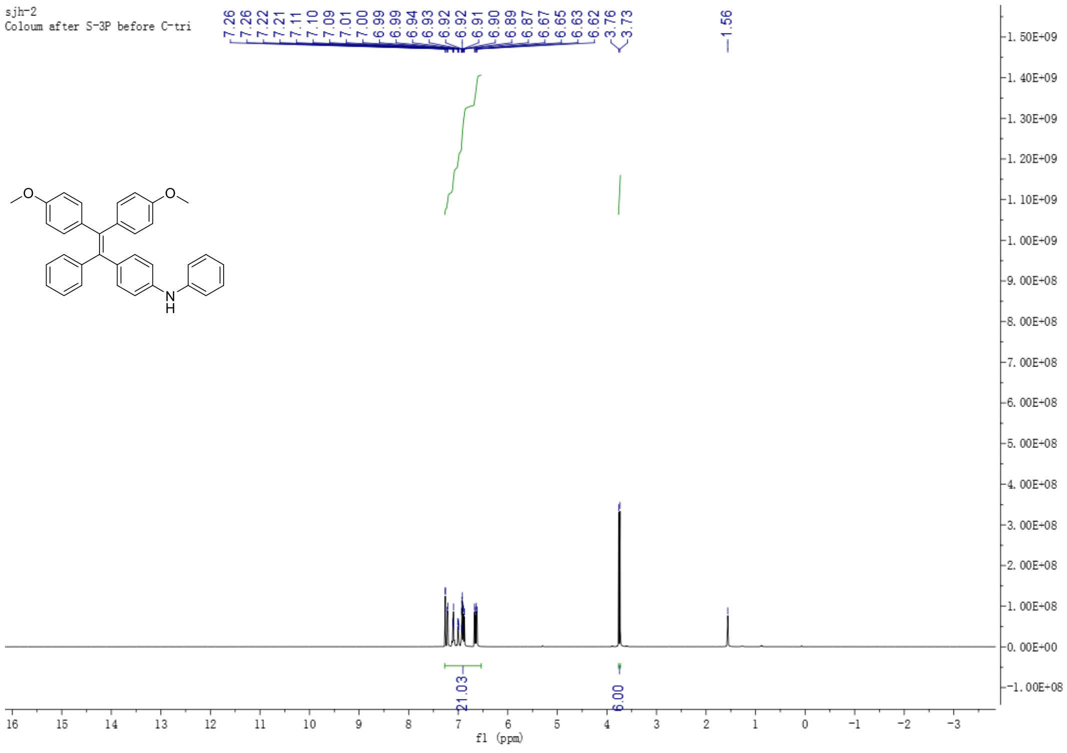

^{1}H NMR Spectra of intermediate 1 (CDCl3, 298 K) Purity= 99%

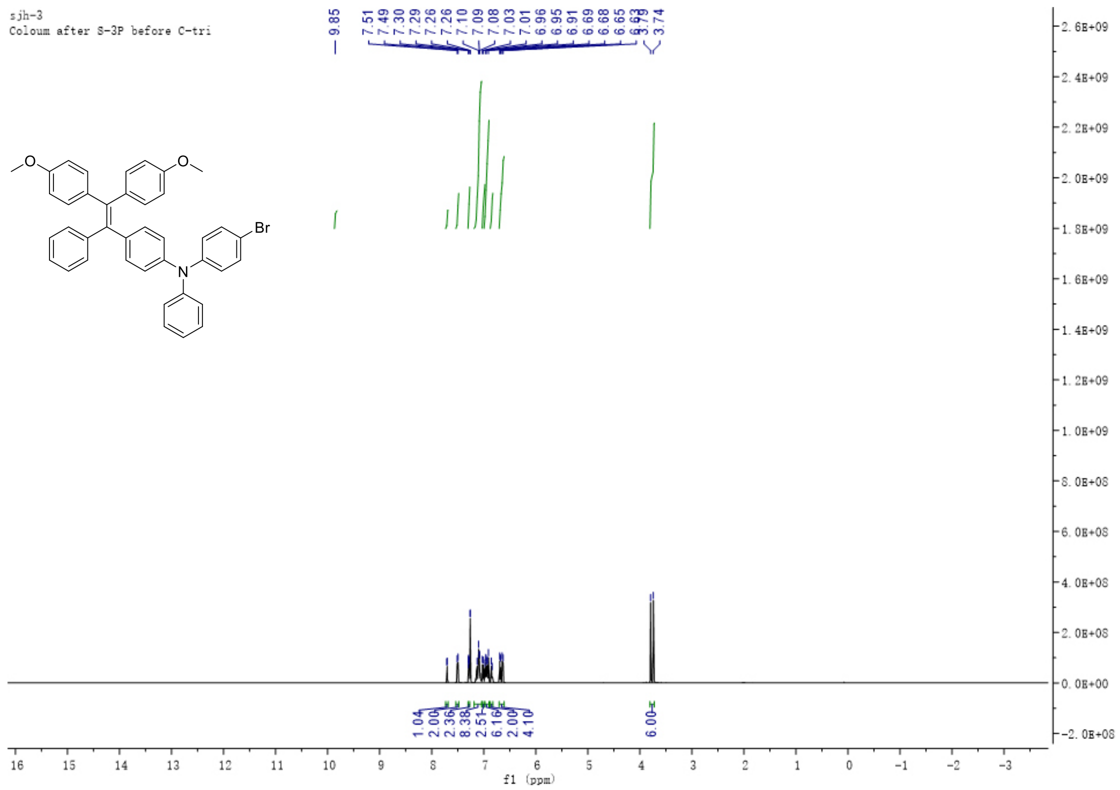

^{1}H NMR Spectra of intermediate 2(CDCl3, 298 K) Purity= 100%

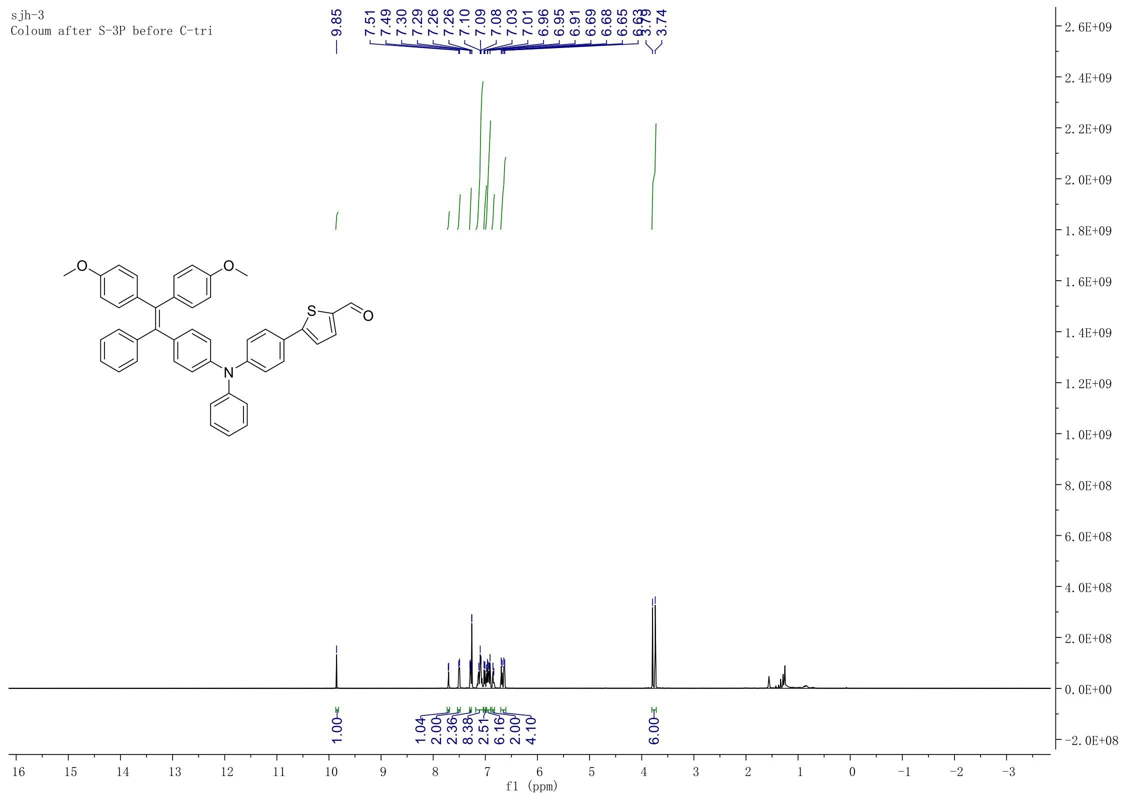

^{1}H NMR spectrum of intermediate 3 (CDCl3, 298 K) Purity= 93 %

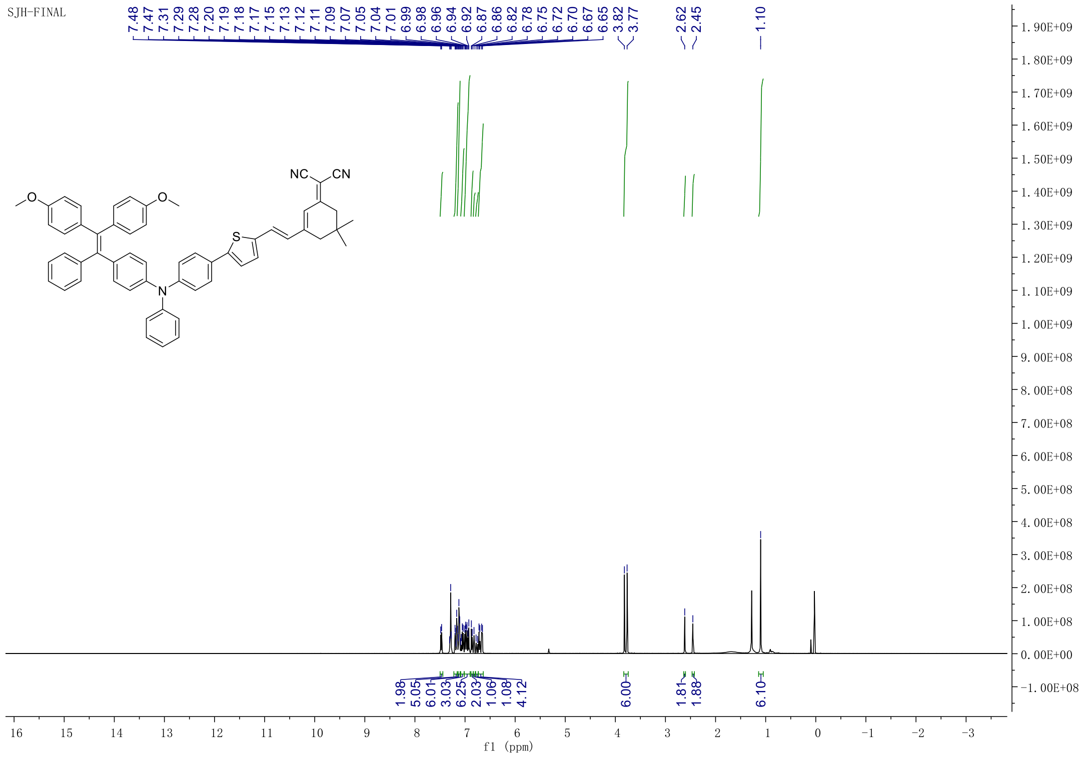

^{1}H NMR spectrum of TTTDM (CDCl3, 298 K) purity=99.95%

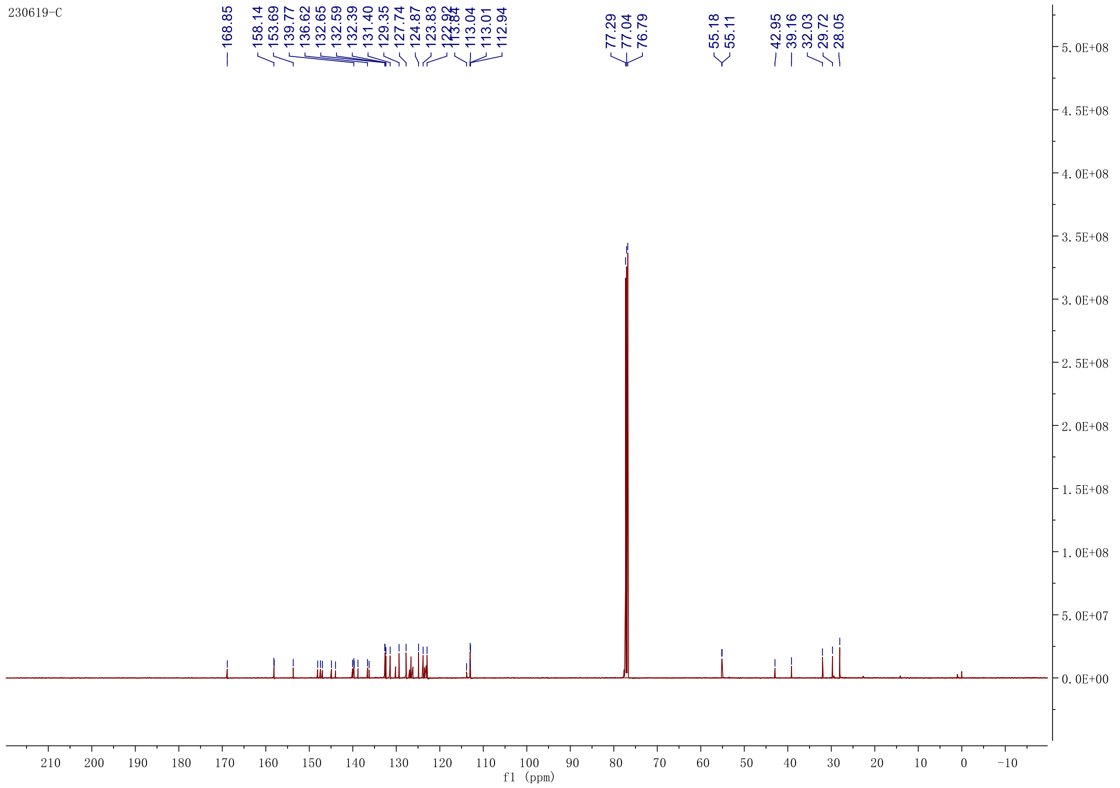

^{13}C NMR spectrum of TTTDM (CDCl3, 298 K)

Mass spectrum of TTTDM

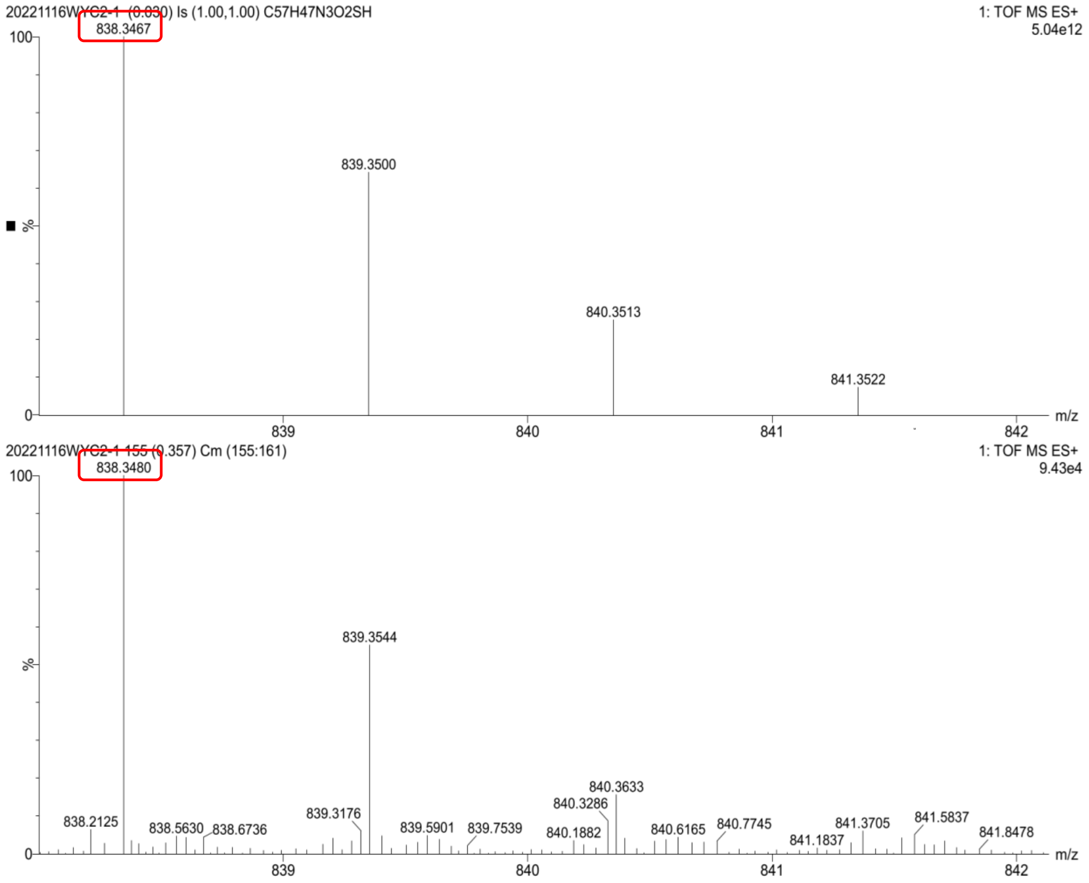

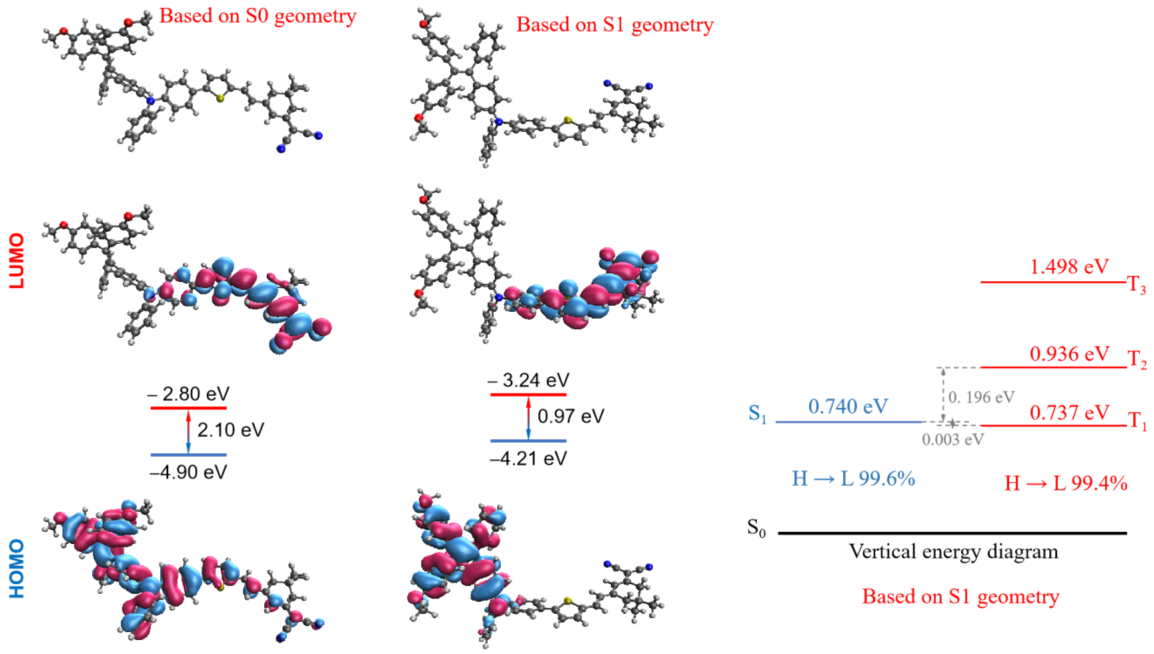

Figure S1 Theoretical calculation

Computational details

The ground-state geometries of the molecule was optimized using density functional theory (DFT) method at the theoretical level of B3LYP-D3/6-31g(d,p). Time-dependent DFT (TD-DFT) method was utilized at the same level of theory to calculate energy levels of singlet, triplet states and their gap (E) based on the optimized singlet-state geometries.[1] Above quantum chemical calculations were carried out by using Gaussian 16 program (Revision A.03).[2] The HOMO and LUMO were displayed using IQmol molecular viewer package (Vision 3.0.1).

[1] C. Adamo and D. Jacquemin. The calculations of excited-state properties with Time-Dependent Density Functional Theory. Chem. Soc. Rev. 42 (2013), 845.

[2] M. J. Frisch, G. W. Trucks, H. B. Schlegel, G. E. Scuseria, M. A. Robb, J. R. Cheeseman, G. Scalmani, V. Barone, G. A. Petersson, H. Nakatsuji, X. Li, M. Caricato, A. V. Marenich, J. Bloino, B. G. Janesko, R. Gomperts, B. Mennucci, H. P. Hratchian, J. V. Ortiz, A. F. Izmaylov, J. L. Sonnenberg, D. Williams-Young, F. Ding, F. Lipparini, F. Egidi, J. Goings, B. Peng, A. Petrone, T. Henderson, D. Ranasinghe, V. G. Zakrzewski, J. Gao, N. Rega, G. Zheng, W. Liang, M. Hada, M. Ehara, K. Toyota, R. Fukuda, J. Hasegawa, M. Ishida, T. Nakajima, Y. Honda, O. Kitao, H. Nakai, T. Vreven, K. Throssell, J. A. Montgomery, Jr., J. E. Peralta, F. Ogliaro, M. J. Bearpark, J. J. Heyd, E. N. Brothers, K. N. Kudin, V. N. Staroverov, T. A. Keith, R. Kobayashi, J. Normand, K. Raghavachari, A. P. Rendell, J. C. Burant, S. S. Iyengar, J. Tomasi, M. Cossi, J. M. Millam, M. Klene, C. Adamo, R. Cammi, J. W. Ochterski, R. L. Martin, K. Morokuma, O. Farkas, J. B. Foresman, and D. J. Fox, Gaussian 16, Gaussian, Inc., Wallingford CT, 2016.

Digital photos of biofilm samples

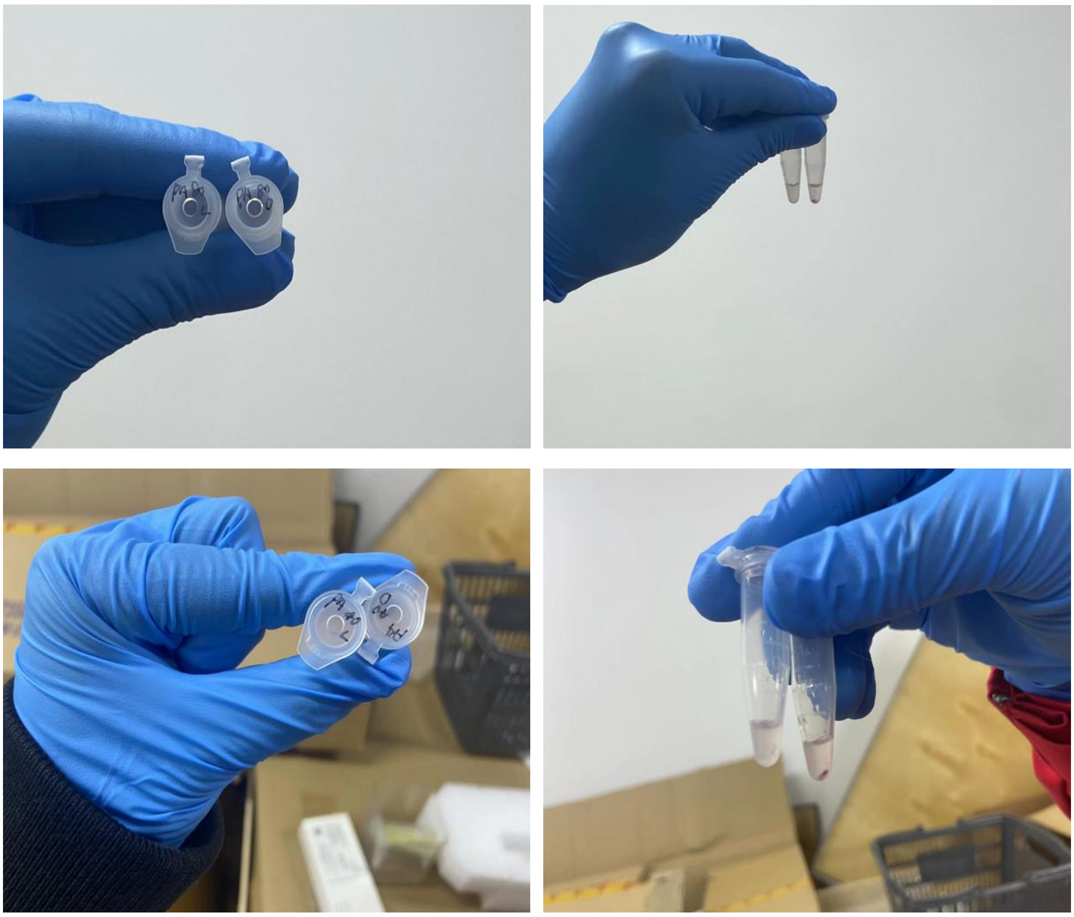

Figure S2. Digital photos of biofilm samples

SEM

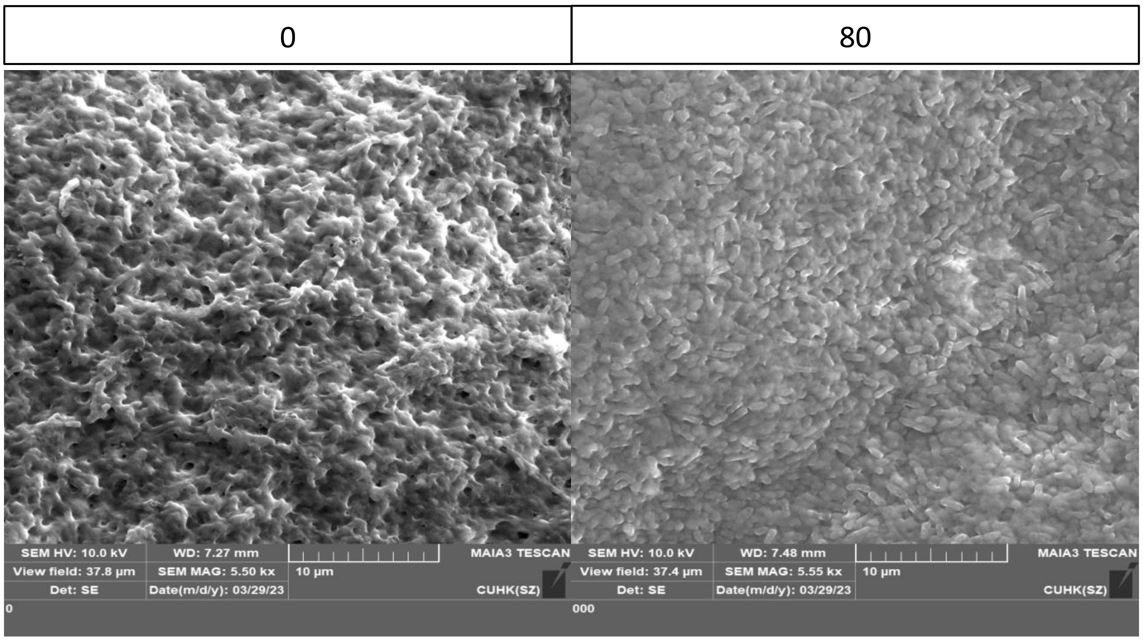

Figure S3. SEM images of P.aeruginosa biofilms after treatment with different concentrations under light conditions (white light 30 mW cm^{-2} for 1 h).
